# Mobile Phone, Computer, and Internet Use Among Older Homeless Adults: Results from the HOPE HOME Cohort Study

**DOI:** 10.2196/10049

**Published:** 2018-12-10

**Authors:** Maria C Raven, Lauren M Kaplan, Marina Rosenberg, Lina Tieu, David Guzman, Margot Kushel

**Affiliations:** 1 Department of Emergency Medicine School of Medicine University of California, San Francisco San Francisco, CA United States; 2 Philip R. Lee Institute for Health Policy Studies University of California, San Francisco San Francisco, CA United States; 3 Center for Vulnerable Populations Zuckerberg San Francisco General Hospital and Trauma Center University of California, San Francisco San Francisco, CA United States; 4 Division of General Internal Medicine Department of Medicine, Zuckerberg San Francisco General Hospital and Trauma Center University of California, San Francisco San Francisco, CA United States; 5 School of Public Health University of California, Berkeley Berkeley, CA United States

**Keywords:** homelessness, internet, cell phone, smartphone, aged and middle aged

## Abstract

**Background:**

The median age of single homeless adults is approximately 50 years. Older homeless adults have poor social support and experience a high prevalence of chronic disease, depression, and substance use disorders. Access to mobile phones and the internet could help lower the barriers to social support, social services, and medical care; however, little is known about access to and use of these by older homeless adults.

**Objective:**

This study aimed to describe the access to and use of mobile phones, computers, and internet among a cohort of 350 homeless adults over the age of 50 years.

**Methods:**

We recruited 350 participants who were homeless and older than 50 years in Oakland, California. We interviewed participants at 6-month intervals about their health status, residential history, social support, substance use, depressive symptomology, and activities of daily living (ADLs) using validated tools. We performed clinical assessments of cognitive function. During the 6-month follow-up interview, study staff administered questions about internet and mobile technology use. We assessed participants’ comfort with and use of multiple functions associated with these technologies.

**Results:**

Of the 343 participants alive at the 6-month follow-up, 87.5% (300/343) completed the mobile phone and internet questionnaire. The median age of participants was 57.5 years (interquartile range 54-61). Of these, 74.7% (224/300) were male, and 81.0% (243/300) were black. Approximately one-fourth (24.3%, 73/300) of the participants had cognitive impairment and slightly over one-third (33.6%, 100/300) had impairments in executive function. Most (72.3%, 217/300) participants currently owned or had access to a mobile phone. Of those, most had feature phones, rather than smartphones (89, 32.1%), and did not hold annual contracts (261, 94.2%). Just over half (164, 55%) had ever accessed the internet. Participants used phones and internet to communicate with medical personnel (179, 64.6%), search for housing and employment (85, 30.7%), and to contact their families (228, 82.3%). Those who regained housing were significantly more likely to have mobile phone access (adjusted odds ratio [AOR] 3.81, 95% CI 1.77-8.21). Those with ADL (AOR 0.53, 95% CI 0.31-0.92) and executive function impairment (AOR 0.49; 95% CI 0.28-0.86) were significantly less likely to have mobile phones. Moderate to high risk amphetamine use was associated with reduced access to mobile phones (AOR 0.27, 95% CI 0.10-0.72).

**Conclusions:**

Older homeless adults could benefit from portable internet and phone access. However, participants had a lower prevalence of smartphone and internet access than adults aged over 65 years in the general public or low-income adults. Participants faced barriers to mobile phone and internet use, including financial barriers and functional and cognitive impairments. Expanding access to these basic technologies could result in improved outcomes.

## Introduction

### Background

In the past 25 years, the median age of individuals experiencing homelessness in the United States has risen [1]. Approximately half of the single adult homeless population is aged 50 years and older [1]. Adults with a current or recent experience of homelessness (homeless-experienced) have a high prevalence of chronic disease, functional and cognitive impairment, and substance use [2-4]. Homeless-experienced older adults’ competing needs for food and shelter, lack of stable mailing address, and limited social support complicate the receipt of longitudinal health care needed to manage these conditions [2,5].

Appropriate longitudinal health care relies on intervisit communication [6-9]. Mobile phones, email, and patient portals increase the consistency of intervisit communication between patients and clinicians and improve self-management of chronic diseases in the general population [5,10-14]. None of these requires a permanent address, and therefore, they could be used by people experiencing homelessness [11,15,16].

In addition to improving health care communication, these technologies have other potential health benefits for homeless individuals, including decreasing social isolation, connecting to social services, and identifying housing resources [17-21]. However, little is known about how older homeless-experienced adults use mobile and internet technologies.

Low-income housed individuals report barriers to technology use, such as lack of high-speed broadband access, limited English proficiency, and limited digital and linguistic literacy [14,22,23]. Low-income populations rely on smartphones, rather than computers, for internet access [14]. Older adults in the general population use technology at lower rates than younger adults [24,25]. Cost; low digital literacy; and cognitive, executive, and sensory impairments may limit use in this population [24-27].

### Objectives

The limited literature about mobile phone and internet access among homeless populations has focused on younger populations [18,28]. Little is known about the use of mobile phones and internet by older adults who experience homelessness. In a population-based cohort of 350 homeless- experienced adults aged 50 years and older, we examined the prevalence of mobile phone (smartphones and feature phones), computer and internet access, purposes of use, types of service contracts and charging locations, and the factors associated with access to mobile phones.

## Methods

### Participants and Setting

The overall goal of the Health Outcomes of People Experiencing Homelessness in Older Middle Age (HOPE HOME) study is, among older homeless adults, to describe the life course events and level of geriatric conditions and to explore the association between life course events and geriatric conditions with acute health service utilization. Between July 2013 and June 2014, we used population-based sampling to recruit 350 homeless individuals aged 50 years or older in Oakland, California [29]. We recruited from homeless encampments, all overnight homeless shelters that served single adults over the age of 25 years (n=5), one recycling center close to homeless service agencies, and all free and low-cost meal programs serving at least 3 prepared meals a week (n=5). We constructed our sampling frame to approximate the source population; we randomly selected potential participants at each recruitment site [30,31].

After an initial screening for eligibility, we invited individuals to complete a detailed eligibility interview within 1 week. Participants were eligible for the study if they were English-speaking, aged 50 years or older, defined as homeless by the Homeless Emergency Assistance and Rapid Transition to Housing Act (HEARTH), [32] and able to give written informed consent as determined by a teach-back method [33]. We gave participants gift certificates worth US $25 for completing the screening and baseline interviews and US $20 for each semiannual follow-up visit. We gave participants a US $5 gift certificate for each monthly check-in between scheduled visits. The majority of study activities took place at St Mary’s Center, a nonprofit community-based organization serving low-income older adults. The institutional review board of the University of California, San Francisco approved the study.

**Figure 1:**
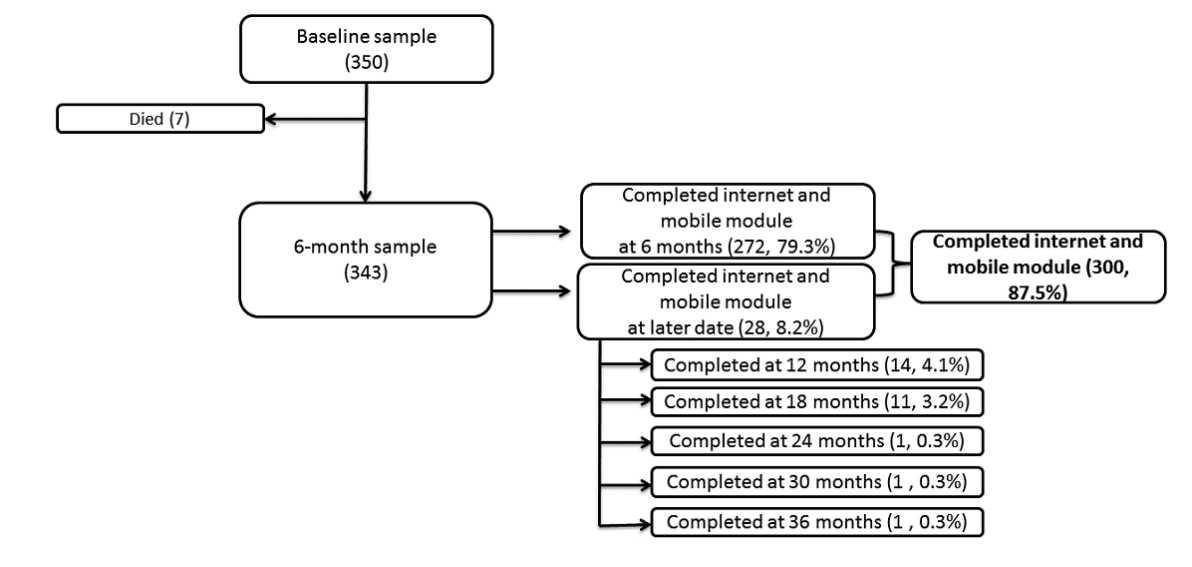
Recruitment flowchart.

Trained study staff administered structured baseline interviews and follow-up interviews at 6-month intervals. At the initial interview, study staff collected extensive contact information on participants, including a phone number, if the participant had one. Participants checked in monthly between study visits, by phone or in person, to enhance the follow-up process. During structured interviews at baseline and follow-up, participants reported information about housing history, demographic information, health history, health care utilization, drug and alcohol use, mental health, and social support, and completed assessments of functional and cognitive impairment. Participants remained in the study independent of their housing status at the time of follow-up. During the 6-month follow-up interview or, if missed, the next attended interview, study staff administered a module centered on the use of internet and mobile technology, as shown in Figure 1.

In this analysis, we use all time-varying variables at the interview at which the participant completed the internet and mobile technology module. To assess differential loss to follow-up, we assessed whether participants who were eligible for, but did not complete, a mobile phone and internet interview were less likely to report having a phone number at enrollment than those who completed the interview.

### Measures

#### Demographics

Demographic variables included age, sex, and race or ethnicity (black, white, Hispanic or Latino, Asian, other or mixed). We dichotomized participants as having completed a high school or General Educational Development (GED) degree versus having completed less than a high school equivalent degree. Participants reported their total income in the past 30 days, categorized as US $0-$150, $151-$700, $701-$1150, and over $1150. To assess health literacy, we used a validated one-item health literacy screen “How confident are you filling out medical forms by yourself?” (Not at all, A little bit, Somewhat, Quite a bit, Extremely) [34]. On the basis of validation studies within low-income populations, we considered those who reported being somewhat confident or less as having limited health literacy [35,36].

### Focal Variables

#### Focal Independent Variable: Housing Status

At each interview, we determined whether participants still met the HEARTH criteria for homelessness, categorizing the participants’ current living situation as homeless, housed, or in an institution. As participants were either currently homeless, or had recently been homeless, and in keeping with the transient nature of homelessness, we described the sample as homeless-experienced [14].

#### Mobile Phone Access, Use, and Service Type

Participants reported if they had ever used a mobile phone (feature, smartphone, or both). We adapted Pew survey items based on prior research on information technologies among homeless populations [37,38]. We defined feature phones as phones allowing users to “make and receive phone calls and text messages, take pictures and perform basic Web browsing.” We defined smartphones as “a phone with a larger screen that allows functions like a mini computer and lets you check your email and use a number of different applications.” We asked participants if they had ever used a mobile phone; if yes, we asked whether they had current access to a mobile phone or had access in the past. We defined having access to a mobile phone as owning a mobile phone, borrowing one long term, being able to borrow one if needed, or being able to find one in an emergency. Participants reported whether they had current access to mobile phones, past access, or never had access. Our focal dependent variable was current access to a mobile phone.

We asked participants to report what type of mobile service they used (contract, month-to-month, prepaid, free phone, or other). If participants reported ever having access to a mobile phone, we asked them to report what they used it for (making phone calls, receiving phone calls, voicemail, or text messages). If participants had ever used a smartphone, we asked them if they used it to check and send email, access social networking sites, look up information on the internet, look up bus schedules, or get directions. We asked participants to report whether they used a mobile phone to contact others, and if so, whom they contacted. We asked participants if they had ever had their mobile phones stolen. If participants reported having had their phones stolen, we asked them how many times. We asked participants if they had ever lost a mobile phone. If they reported losing a phone, we asked how many times.

##### Ease of Use and Charging Locations

We asked participants to report, on a 5-point Likert scale (1=very easy to 6=I don’t know how to do this), how comfortable they were with performing the following actions on a mobile phone: making a call, answering a call, contacting 911 or emergency medical services, checking voicemail, and using text messaging. Participants rated the difficulty of using basic components of their phone, such as the buttons and screen. We asked participants where they charged their phones. To assess barriers to phone charging, we asked participants whether there were times they had not had mobile phone service because they did not have a place to charge their phones.

##### Computer, Internet, and Email Use

We asked participants if they had ever used a computer. If so, we asked if they had ever used the internet. We asked those who had ever used the internet if they had done so in the past 30 days. Among those with recent use, we asked where they used the internet and what they used the internet for. Potential venues included the following: on a mobile phone, in a public or university library, drop-in center or shelter, friend or relative’s house, internet café, coffee shop or restaurant, social service agency, motel or hotel lobby, church, and others. Uses included reading or sending email; getting news online; watching a video, downloading a music file or playing a game; browsing the internet for fun; searching for a fact or to answer a question; looking for information about a shelter or place to live, a hobby or interest, health or medical information, or about a job; checking social networking sites; doing research for school, training, or education; sending instant messages; refilling a prescription; and looking for a sex partner. For each of these response categories, we asked participants to note all those that were applicable.

We asked participants whether they had ever used email and if they had a current email account. We asked what they used their email for: staying in touch with family or friends, job searches, housing searches, staying in touch with a case manager or social worker, staying in touch with a health care provider, and other. We asked participants to note all that were applicable.

#### Descriptive Variables

##### Health History

We asked participants to rate their health status, dichotomized as poor or fair versus good, very good, or excellent [39]. On the basis of the National Health and Nutrition Examination Survey, we asked participants to report whether a health care provider had ever told them they had any of the 10 chronic conditions [3,40]. We created a composite variable for the total number of chronic conditions, categorized as none, 1, 2, or 3 or more. We asked participants if they had difficulty performing any activities of daily living (ADL): dressing, bathing or showering, eating, getting in or out of bed, or using the toilet [41]. We dichotomized participants as having any difficulty versus no ADL difficulty.

We administered the Modified Mini-Mental (3MS) Examination to assess global cognitive impairment [42] and the Trail Making Test B (Trails B) [43] to assess executive function. Comparing scores with age- and education-adjusted reference values, we categorized scores below the seventh percentile on the 3MS as cognitive impairment [44]. We classified the participants’ performance as “unable to complete” if their time to complete the Trails B lasted longer than 5 min. We interpreted scores with the demographically adjusted (age, gender, and race or ethnicity) norms for the Halstead-Reitan Neuropsychological Test Battery, which uses the Halstead-Reitan Battery (HRB) Norms scoring program [45].

##### Mental Health, Substance Use, and Social Support

We assessed depressive symptoms using the Center for Epidemiologic Studies Depression Scale (CES-D) [46]. On the basis of past studies with older adults, we classified scores of ≥22 as indicative of major depressive symptoms [47,48]. We considered participants who reported drinking ≥6 drinks on 1 occasion every month as heavy drinkers [49]. Using the World Health Organization’s Alcohol, Smoking, and Substance Involvement Screening Test (ASSIST) with a lengthened time frame of the past 6 months, we assessed illicit drug use for cocaine, opioids, or amphetamines [50]. We classified scores of ≥4 for any illicit drug as moderate to high risk use of an illicit substance. To assess social support, we asked participants how many close friends or relatives they had in whom they could confide (0, 1-5, or 6 or more) [51,52].

### Analysis

We performed a descriptive analysis to assess the prevalence of mobile phone, computer, internet, and email use and the purposes of usage. To identify facilitators to mobile phone and internet use among older homeless adults, we assessed the ease of use, types of service, and charging locations. We assessed bivariable associations between current mobile phone use and a priori independent variables using logistic regression. We built our multivariable model by including variables with bivariable type 3 *P* values <.20, and reduced the model using backwards elimination retaining variables with *P* values <.05 for our final multivariable model. We implemented our models in SAS 9.4 (SAS Institute, Cary, NC).

## Results

### Follow-Up

Of the 350 individuals enrolled in the study, 7 died before the 6-month follow-up. Of the 343 participants alive by the 6-month follow-up, 300 (87.5% (300/343) completed the module on internet and mobile phone use. Of these, 79.3% (272/343) completed the mobile phone and internet module at 6 months and 8.2% (28/343) completed the module at a later date. (Figure 1). One-third (32.6%, 14/43) of those who were eligible but did not complete a mobile phone interview reported having a phone number at enrollment, compared with 68.0% (204/300) of those who completed the interview (*P*<.001).

### Demographics

The median age of participants was 57.5 years (interquartile range 54-61). Most participants (74.7%) were male and black (81.0%; see Table 1). Approximately one-fourth had less than a high school or equivalent (eg, GED) education (24.7%). Most participants (74.3%) remained homeless at their follow-up interview. Approximately three-fourths (74.9%) reported having at least one confidant. Over ten percent (10.3%) reported heavy drinking, and approximately one-third met the criteria for moderate- to high-risk cocaine use (29.0%).

**Table 1:** Participant characteristics of mobile phone use.

Descriptive, health, and health-related variables		Total (N=300)	Currently own or have access to a mobile phone (N=217)	Owned or have access to a mobile phone (N=60)	Never owned or had access to a mobile phone in the past (N=23)
Age in years, median (interquartile range)		57.5 (54.0-61.0)	57.0 (54.0-61.0)	58.0 (54.0-61.0)	58.0 (55.0-65.0)
Male, n (%)		224 (74.7)	161 (74.2)	44 (73)	19 (83)
Black, n (%)		243 (81.0)	183 (84.3)	45 (75)	15 (65)
Completed less than high school degree^a^, n (%)		74 (24.5)	57 (26.2)	10 (177)	7 (30)
**Total income in past 30 days, n (%)**					
	US $0-150	62 (20.6)	41 (18.8)	13 (22)	8 (35)
	US $151-700	76 (25.3)	55 (25.3)	19 (32)	2 (9)
	US $701-1150	128 (42.7)	92 (42.3)	24 (40)	12 (52)
	More than US $1150	34 (11.3)	29 (13.3)	4 (7)	1 (4)
Homeless at follow-up interview^b^		223 (74.3)	149 (68.7)	55 (92)	19 (83)
Social support, n (%)		224 (74.9)	169 (77.9)	40 (67)	15 (68)
**Number of confidants^c^**					
	None	76 (25.3)	49 (22.5)	20 (33)	7 (32)
	1	78 (26.0)	55 (25.3)	17 (28)	6 (27)
	2	46 (15.3)	33 (15.2)	9 (15)	4 (18)
	3 or more	99 (33.0)	80 (36.8)	14 (23)	5 (23)
Fair or poor health, n (%)		166 (56.1)	113 (52.8)	39 (66)	14 (61)
**Number of chronic conditions, n (%)**					
	None	76 (25.3)	58 (26.7)	13 (22)	5 (22)
	1	103 (34.3)	76 (35.0)	18 (30)	9 (39)
	2	88 (29.3)	61 (28.1)	19 (32)	8 (35)
	3 or more	33 (11.0)	22 (10.1)	10 (17)	1 (4)
Activities of daily living impairment, n (%)		128 (42.7)	85 (39.1)	31 (52)	12 (52)
Cognitive impairment (3MS, baseline)^d^, n (%)		73 (24.3)	46 (21.1)	17 (28)	10 (44)
Executive function impairment (Trails B)^e^, n (%)		100 (33.6)	65 (29.9)	27 (45)	8 (35)
Moderate-to-severe depressive symptoms^f^, n (%)		94 (31.3)	63 (29.0)	22 (37)	9 (43)
Heavy drinking^g^, n (%)		31 (10.3)	16 (7.3)	11 (4)	3 (13)
Moderate-to-high risk amphetamines use^h^, n (%)		19 (6.3)	8 (3.6)	9 (15)	2 (9)
Moderate-to-high risk cocaine use^i^, n (%)		87 (29.0)	59 (27.1)	20 (33)	8 (35)
Moderate-to-high risk opioids use^j^, n (%)		19 (6.3)	12 (5.5)	5 (8)	2 (9)

^a^Completion of high school degree included General Education Development (GED).

^b^Homeless as defined by the Homeless Emergency Assistance and Rapid Transition to Housing (HEARTH) Act.

^c^Confidant defined as “a close friend or family member in whom you can confide or talk about yourself and your problems.”

^d^Modified Mini-Mental State Examination; less than seventh percentile based on Z-scores used.

^e^Trail Making Test; more than 5-min completion time on Trails B.

^f^Score of ≥22 on the Center for Epidemiologic Studies Depression Scale (CES-D).

^g^Drinking ≥6 drinks on one occasion every month.

^h^Score of ≥4 for any amphetamines using the World Health Organization’s Alcohol, Smoking, and Substance Involvement Screening Test (ASSIST).

^i^Score of ≥4 for any cocaine using the World Health Organization’s Alcohol, Smoking, and Substance Involvement Screening Test (ASSIST).

^j^Score of ≥4 for any opioids using the World Health Organization’s Alcohol, Smoking, and Substance Involvement Screening Test (ASSIST).

Approximately one-third (31.8%) met the criteria for major depressive symptoms. Over half (56.1%) described their health as fair to poor and approximately three-fourths had at least one chronic condition (74.7%). Over 40% had ADL impairment (42.7%), approximately one-fourth had cognitive impairment (24.3%), and one-third had problems in executive functioning (33.6%).

### Mobile Phone Access, Use, and Service Type

Almost all participants currently owned or had access to a mobile phone (72.3%) or had owned or had access to a mobile phone in the past (20.0%; see Table 2). Among those with current mobile phone access, 204 owned their phones, 4 borrowed phones for long term, 6 borrowed phones for short term, and 3 could access a mobile phone in an emergency. Among participants who currently or had ever had a phone (n=277), two-thirds had basic mobile phones, as opposed to smartphones.

More than three-fourths of participants with current or prior access to a phone (n=277) reported using phones to contact relatives (82.3%) and friends (77.6%; see Table 2). A majority of participants used phones to contact medical personnel (66.6%), and nearly half of them used phones to contact social service agencies (49.5%). Almost one-third used phones to contact shelters or other housing providers (30.7%). Approximately one-fourth used them to contact potential employers (23.1%). A smaller proportion used phones to contact potential landlords (19.5%; see Table 2). Over half reported having had their mobile phones stolen (53.1%) or lost (52.9%).

### Ease of Use and Charging Locations

Over 80% of participants with experience with mobile phones reported that it was easy to use them (Table 3). Participants reported charging their phones at a variety of locations, most commonly at a relative or friend’s (34.3%) or a drop-in center or shelter (32.5%; see Table 3). Over half (56.1%) of those with past access to mobile phones versus approximately one-third (31.6%) of those with current mobile phone access reported not having service due to not having a place to charge their phones.

### Computer and Internet Use

A majority of the participants reported using a computer (64.8%) or accessing the internet (55.0%) during their lifetime (see Table 4). Approximately one-third of the participants had used a computer (37.9%) or the internet (39.3%) in the past 30 days. Participants accessed the internet from a variety of locations, most of which were public. They used the internet for multiple functions including email (24.8%) and looking for information about housing (16.8%), medical information (15.1%), or a job (14.4%; see Table 4).

Approximately one-third had a current email account (35.2%). The most common uses of email were staying in touch with family or friends and searching for jobs and housing (Table 4).

### Factors Associated With Mobile Phone Access

In an adjusted multivariable regression model (see Table 5), we found that individuals who were housed at the time of this interview had 3.81 (adjusted odds ratio [AOR] 3.81, 95% CI 1.77-8.21) higher odds of currently owning a mobile phone, compared with those who were not housed (see Table 5). Moreover, 3 factors were associated with significantly lower odds of current mobile phone ownership: ADL impairment (AOR 0.53, 95% CI 0.31-0.92), executive function impairment (AOR 0.49, 95% CI 0.28-0.86), and moderate to high use of amphetamines (AOR 0.27, 95% CI 0.10-0.72).

**Table 2:** Mobile phone use.

Mobile phone use		Total (N=277), n (%)	Currently own or have access to a mobile phone (N=217), n (%)	Owned or had access to a mobile phone in the past (N=60), n (%)	*P* value
**Type of phone use or used**					
	Feature phone	186 (67.1)	143 (65.9)	43 (72)	.57
	Smartphone	89 (32.1)	72 (33.1)	17 (28)	—^a^
	Both	2 (0.7)	2 (0.9)	0 (0)	—
**Type of service**					
	Contract	16 (5.7)	13 (5.9)	3 (5)	.10
	Month-to-month	167 (60.3)	130 (59.9)	37 (62)	—
	Free phone	52 (18.7)	46 (21.1)	6 (10)	—
	Prepaid	26 (9.3)	16 (7.3)	10 (17)	—
	Other or don’t know	16 (5.7)	12 (5.5)	4 (7)	—
**Mobile phone features used**					
	Make and receive phone calls	277 (100.0)	217 (100.0)	60 (100)	>.99
	Check and receive voicemails	195 (70.4)	162 (74.7)	33 (55)	.003
	Send and receive text messages	172 (62.9)	145 (66.8)	27 (45)	.002
**Smartphone features used**					
	Look up information on the internet	66 (23.8)	55 (25.3)	11 (65)	.42
	Check and send email	53 (19.1)	46 (21.1)	7 (41)	.11
	Get directions	49 (17.6)	41 (18.8)	8 (47)	.53
	Look up bus route or schedule	37 (13.3)	34 (15.6)	3 (18)	.03
	Check social networking sites	24 (8.6)	23 (10.5)	1 (6)	.03
**Uses of phone to contact others**					
	Use or used phone to contact relatives	228 (82.3)	178 (82.0)	50 (83)	.81
	Use or used phone to contact friends	215 (77.6)	171 (78.8)	44 (73)	.37
	Use or used phone to contact medical personnel	179 (64.6)	150 (69.1)	29 (48)	.003
	Use or used phone to contact social service agencies	137 (49.5)	115 (53.0)	22 (37)	.03
	Use or used phone to contact shelters or other housing providers	85 (30.6)	71 (32.7)	14 (23)	.16
	Use or used phone to contact (potential) employer	64 (23.1)	51 (23.5)	13 (22)	.77
	Use or used phone to contact (potential) landlord	54 (19.4)	44 (20.2)	10 (17)	.53
	Use or used phone to contact emergency services	29 (10.4)	25 (11.5)	4 (7)	.28
Ever had phone stolen		146 (53.1)	106 (49.3)	40 (67)	.02
**Number of times phone stolen**					
	0	129 (47.4)	109 (51.2)	20 (344)	.06
	1-2	109 (40.1)	80 (36.8)	29 (49)	.06
	≥3	34 (12.2)	24 (11.0)	10 (17)	.06
Ever lost phone		145 (52.9)	113 (52.6)	32 (54)	.81
**Number of times lost phone**					
	0	129 (47.4)	102 (47.9)	27 (46)	.30
	1-2	106 (39.0)	79 (36.4)	27 (46)	.30
	≥3	37 (12.2)	32 (14.7)	5 (9)	.30

^a^Not applicable.

**Table 3:** Ease of using mobile phone features and charging among participants who had ever used a mobile phone (N=277).

Ease of use and charging locations		Total (N=277), n (%)	Currently own or have access to a mobile phone, (N=217), n (%)	Owned or had access to a mobile phone in the past, (N=60), n (%)	*P* value
**Proportion reporting very easy to neither easy nor difficult, n (%)**					
	Punching buttons on the screen	240 (86.6)	191 (88.0)	49 (82)	.20
	Seeing the phone screen	217 (78.3)	174 (80.2)	43 (72)	.16
	Hearing the phone ring	239 (86.3)	189 (87.1)	50 (83)	.45
	Hearing people talk	222 (80.1)	179 (82.5)	43 (72)	.06
	Using voicemail	191 (69.0)	160 (73.7)	31 (52)	.001
	Using other mobile phone features (eg, contacts)	198 (71.5)	167 (77.0)	31 (52)	<.001
**Charging locations**					
	A friend or relative’s house	95 (34.2)	81 (37.3)	14 (23)	.04
	A drop-in center or homeless shelter	90 (32.4)	70 (32.2)	20 (33)	.87
	A library	37 (13.3)	25 (11.5)	12 (20)	.09
	A coffee shop or restaurant	39 (14.0)	24 (11.0)	15 (25)	.01
	A city power supply	19 (6.8)	9 (4.1)	10 (17)	<.001
	A social service or case management agency	18 (6.4)	13 (5.9)	5 (8)	.51
	A place where you pay to charge your phone	3 (1.0)	1 (0.5)	2 (3)	.06
No service due to lack of a charging location^a^		98 (35.3)	66 (30.4)	32 (56)	<.001

^a^11 participants had missing data.

**Table 4:** Computer, internet, and email use.

Computer, internet, and email use		n (%)
Ever used a computer^a^		193 (64.8)
Currently use a computer		113 (37.9)
Ever used internet		164 (55.0)
Used internet, last 30 days		117 (39.3)
**Venues where internet was used^b^**		
	Public or university library	59 (19.8)
	On mobile phone	51 (17.1)
	Drop-in center or homeless shelter	27 (9.1)
	Friend or relative’s house	21 (7.0)
	Internet café, coffee shop, or restaurant	11 (3.4)
	Social service agency	8 (2.7)
	Motel or hotel lobby	3 (1.0)
	Church	1 (0.3)
	Workplace	2 (0.7)
	Other venue	26 (8.7)
**Purpose of using the internet**		
	Read or send email	74 (24.8)
	Get news online	66 (22.1)
	Watch a video, download a music file, or play a game	61 (20.5)
	Browse the internet for fun	56 (18.8)
	Search for a fact or to answer a question	50 (16.8)
	Look for information about a shelter or place to live	50 (16.8)
	Look for information about a hobby or interest	46 (15.4)
	Look for health or medical information	45 (15.1)
	Look for information about a job	43 (14.4)
	Check social networking sites (eg, Facebook or Twitter)	43 (14.4)
	Do research for school or training, or obtain education	25 (8.4)
	Send instant messages	18 (6.0)
	Refill a prescription	16 (5.4)
	Look for a (sex) partner	4 (1.3)
**Email**		
	Know what email is	234 (78.5)
	Know how to use email	144 (48.3)
	Have an email account	105 (35.2)
**Uses of email^c^**		
	Stay in touch with family or friends	67 (22.5)
	Job searches	46 (15.4)
	Housing searches	40 (13.4)
	Stay in touch with health care providers	21 (7.0)
	Stay in touch with case manager or social workers	16 (5.4)
	Other	21 (7.0)

^a^Two participants are not included in the computer and internet use section because they did not report whether they had ever used a computer, N=298

^b^The denominator for internet venues and uses is 117.

^c^The denominator for email variables ranges from 296.3 to 300.

**Table 5:** Odds of current mobile phone use.

Independent variables		Unadjusted odds ratio (95% CI)	*P* value	Adjusted odds ratio (95% CI)	*P* value
Black		2.06 (1.12-3.80)	.02	—^a^	—
Housed^b^		3.75 (1.76-7.99)	<.001	3.81 (1.77-8.21)	<.001
**Health history**					
	Good to excellent health	1.63 (0.96-2.78)	.07	—	—
	ADL impairment^c^	0.60 (0.36-1.00)	.05	0.53 (0.31-0.92)	.02
	Cognitive impairment (3MS, baseline)^d^	0.56 (0.32-0.99)	.04	—	—
	Executive function impairment (Trails B)^e^	0.59 (0.35-1.01)	.05	0.49 (0.28-0.86)	.01
	Heavy drinking^f^	0.58 (0.32-1.02)	.06	—	—
	Moderate to high risk amphetamine use^g^	0.25 (0.10-0.65)	.004	0.27 (0.10-0.72)	.01

^a^Not applicable.

^b^Not homeless as defined by the Homeless Emergency Assistance and Rapid Transition to Housing (HEARTH) Act.

^c^Difficulty performing one or more activities of daily living (ADL): dressing, bathing or showering, eating, getting in or out of bed, or using the toilet.

^d^Modified Mini-Mental State Examination; less than seventh percentile based on Z-scores used.

^e^Trail Making Test; more than 5-min completion time on Trails B.

^f^Drinking ≥6 drinks on one occasion every month.

^g^Score of ≥4 for amphetamine use using the World Health Organization’s Alcohol, Smoking, and Substance Involvement Screening Test (ASSIST).

## Discussion

### Principal Findings

In a sample of homeless-experienced adults aged 50 years and older, almost three-fourths of participants had current access to a mobile phone. Participants with phones used them for social support and communication with health care providers; however, few had annual phone contracts. Without annual contracts, it is likely that participants’ phone numbers changed frequently, limiting the utility for two-way communication.

Although compared with the general population, there was less use of the internet, a high proportion who reported ever having used the internet had used it in the prior 30 days, suggesting that individuals who had knowledge about the internet used it regularly [53]. Participants who did use the internet in the prior month used it to get directions, bus schedules, and to obtain information on employment and housing—all of which can be invaluable for individuals experiencing homelessness [5,54].

When we recruited our participants, all were homeless. A significantly higher proportion of those who were housed at the time of this interview had current access to a mobile phone. Those with current mobile phone access were significantly more likely to use phones to contact shelters or other housing providers than those without current access [55]. In our multivariable analysis, we found that being housed was significantly associated with current mobile phone ownership. A stable living situation may enable more consistent mobile phone ownership, or access to a mobile phone may have helped participants regain housing. Poor executive and cognitive functions and moderate to high risk amphetamine use were negatively associated with current mobile phone ownership. Each of these 3 factors can adversely affect an individual’s ability to participate in the type of anticipatory planning and organization required to obtain and maintain technology—even simple technology such as mobile phones. A majority of participants had lost or had their phones stolen, reflecting one of many adversities in the experience of homelessness.

### Limitations

Our study has several limitations. First, our estimates of mobile phone access were likely overestimates. We introduced the mobile phone and internet module at the first follow-up interview after the baseline interview. There was differential loss to follow-up. Participants without phones at enrollment were less likely to complete the follow-up interview. Second, not all participants remained homeless at the time of the interview; those with housing were more likely to have phones than those without housing. Finally, we used a liberal definition of access to mobile phones, including those who owned or borrowed phones, or had access to one. We relied on participants to self-report mobile phone and internet use and did not have any way to verify these reports with phone bills, direct observation, or other methods.

### Comparison With Prior Work

Participants’ access to mobile phones and the internet was much lower than the general population, of whom 95% own mobile phones (77% of which are smartphones) and 90% of whom use the internet [53]. Participants in our study had lower rates of smartphone and internet use when compared with low-income adults of any age [53,56,57]. Of the minority of participants who were able to access the internet, they accessed it most commonly via smartphones and public libraries. The prevalence of internet access via smartphones in our study was lower than that of those with low incomes in the general population [53]. A majority of participants reported having phones stolen and lost. Having assets stolen or lost is a common experience for people experiencing homelessness. If phones increase the risk of robbery, they may present a safety hazard for older homeless adults.

There are several ways in which older homeless adults could benefit from internet and phone access. Participants used these technologies for health care communication and to seek housing and employment information. Increasing internet and mobile phone access among older homeless adults could allow older homeless adults to more easily apply for housing or to search for housing in areas outside of urban centers that may be lower-cost. Internet and mobile phone access could also facilitate contact with potential employers and increase access to employment and social networking sites.

Mobile phones can facilitate communication with family or friends who may be able to provide instrumental as well as emotional support [17]. Social support has been shown to be associated with better health [58]. In addition, homeless individuals need low-barrier access to outpatient primary care; mobile phones and internet access could facilitate this. A pilot study that examined the feasibility and potential efficacy of using text messages to remind homeless veterans about appointments found that the veterans liked receiving the messages, and those messages may have improved appointment attendance [59]. Two-thirds of our participants reported using their phones to communicate with their health care providers, suggesting both interest and feasibility.

Our participants did not have annual phone contracts. This limited the possibilities for bidirectional communication due to interruptions in service and changing phone numbers. Previous research has cited barriers to mobile phone use among homeless individuals, including cost, fear of loss or theft, and a lack of knowledge about how to use mobile technology [19,37]. The widespread use of month-to-month, instead of annual plans, the use of borrowed (instead of owned) phones, and the relatively low proportion of people who had current access to phones may reflect these barriers, particularly cost. Although there are some programs to address financial barriers to mobile phone use among low-income populations, we found participants had low rates of enrollment in such programs. The “Lifeline” program provides Federal Assistance recipients and those who provide proof of low income with free feature or smartphones and pays for voice calls and texting for a year, with the possibility of recertification [60]. Although most of our participants met the criteria for this program, few reported using its free mobile phones and service. The Lifeline program requires a mailing address. Many people experiencing homelessness lack a stable mailing address, which could cause phone service interruptions.

Without the widespread adoption of phone contracts by homeless adults, health care providers should consider open access scheduling, which could allow homeless adults with any form of phone access to make appointments, while acknowledging their inability to receive appointment reminder calls and texts. Open access scheduling allows for same day appointments and does not rely on reminder calls for appointments scheduled far in advance. This could lower access barriers for individuals experiencing homelessness who may have minimal or no access to mobile phones and the internet. In addition, allowing mobile phone users to maintain the same phone number despite interruptions in service could increase their ability to communicate with health care providers.

Participants in our study did not report difficulty with using phone buttons or keyboard. However, impairments in ADLs and executive function were associated with lower odds of current mobile phone use. Given the levels of these impairments among our participants, more research is needed to match end users with appropriate training tools and technology. It is possible that many who use feature phones could make use of smartphones with appropriate access and training. Others may require improved access and training to make use of feature phone technology.

Another possibility is that impairments in ADLs and executive functioning indirectly decrease use of mobile phones by making it difficult to obtain mobile phones and maintain service. Participants without phones reported a higher likelihood of losing service due to not having a place to charge their phone. Therefore, multipronged approaches that include increasing access to phones, charging stations, and internet might be most effective in increasing the adoption of mobile technology among older adults experiencing homelessness.

Increased public access to high-speed internet and providing discounted smartphones for high-need, low-income individuals may increase access to the internet [61]. Private sector technology and telecommunication companies might also be incentivized to fund initiatives that increase the use of their services among underserved populations, increasing access to reliable mobile technology [61]. Older adults comprise an increasing proportion of the US population. One way for technology companies to increase adoption of mobile phones for older adults is to include them in participatory design and usability testing [62,63]. Adapting devices and tailoring online advanced features to meet the needs of older homeless adults could facilitate their inclusion in the digital economy.

### Conclusions

This study is one of the first studies to examine mobile phone and internet use among a community-based sample of homeless adults over the age of 50 years [64]. The majority of participants with access to technology were able to take advantage of most mobile phone functions, although most of their mobile phones were feature phones with limited internet access. Participants used these technologies for health care communication, seeking information for housing, and looking for employment opportunities.

However, most participants did not have annual phone contracts—which can lead to new phone numbers with each new phone—and few had access to smartphones. Lowering financial barriers to allow annual mobile phone contracts and increasing the homeless individuals’ ability to access the internet via smartphones could promote more reliable and widespread use of these basic technologies. In addition, providers can take steps to optimize the technology individuals experiencing homelessness have access to, by offering open access and same-day scheduling and communication. More research is needed to determine if increasing access to mobile phones and internet can positively impact downstream health and economic outcomes among individuals experiencing homelessness.

The high prevalence of functional and executive function impairment in our study population was negatively associated with access to mobile phones. Advanced technological features might be challenging for this segment of the homeless- experienced population. Initiatives to increase access to technology among older homeless adults must address the needs of those with impairments and create technological features that fit the individuals’ needs and abilities.

## Abbreviations

ADL: activity of daily living
AOR: adjusted odds ratio
ASSIST: Alcohol, Smoking, and Substance Involvement Screening Test
CES-D: Center for Epidemiologic Studies Depression Scale
GED: General Educational Development
HEARTH: Homeless Emergency Assistance and Rapid Transition to Housing
HOPE HOME: Health Outcomes of People Experiencing Homelessness in Older Middle Age
Trails B: Trail Making Test
3MS: Modified Mini-Mental State
